# Flowers as viral hot spots: Honey bees (*Apis mellifera*) unevenly deposit viruses across plant species

**DOI:** 10.1371/journal.pone.0221800

**Published:** 2019-09-18

**Authors:** Samantha A. Alger, P. Alexander Burnham, Alison K. Brody

**Affiliations:** Biology Department, University of Vermont, Burlington, Vermont, United States of America; University of North Carolina at Greensboro, UNITED STATES

## Abstract

RNA viruses, once considered specific to honey bees, are suspected of spilling over from managed bees into wild pollinators; however, transmission routes are largely unknown. A widely accepted yet untested hypothesis states that flowers serve as bridges in the transmission of viruses between bees. Here, using a series of controlled experiments with captive bee colonies, we examined the role of flowers in bee virus transmission. We first examined if honey bees deposit viruses on flowers and whether bumble bees become infected after visiting contaminated flowers. We then examined whether plant species differ in their propensity to harbor viruses and if bee visitation rates increase the likelihood of virus deposition on flowers. Our experiment demonstrated, for the first time, that honey bees deposit viruses on flowers. However, the two viruses we examined, black queen cell virus (BQCV) and deformed wing virus (DWV), were not equally distributed across plant species, suggesting that differences in floral traits, virus ecology and/or foraging behavior may mediate the likelihood of deposition. Bumble bees did not become infected after visiting flowers previously visited by honey bees suggesting that transmission via flowers may be a rare occurrence and contingent on multiplicative factors and probabilities such as infectivity of virus strain across bee species, immunocompetence, virus virulence, virus load, and the probability a bumble bee will contact a virus particle on a flower. Our study is among the first to experimentally examine the role of flowers in bee virus transmission and uncovers promising avenues for future research.

## Introduction

Pathogens are among the top threats to bees causing colony losses, population declines, and a growing concern for food security and ecosystem function [1–4]. Although the importance of pathogens to bees has garnered much attention over the past two decades, there are many unanswered questions regarding the dispersal mechanisms and transmission dynamics of bee pathogens [5]. Numerous pathogens have been detected across broad host ranges including solitary bees, bumble bees, honey bees, ants, wasps, and beetles [6–8]. Shared floral resources, which might act as dispersal platforms between comingling pollinator species, have been implicated in providing transmission routes through which these pathogens may be acquired [5,9–12]. However, few studies have directly examined this route for bee parasites. *Crithidia bombi*, a trypanosome parasite of bumble bees, was transmitted among bumble bees after visiting flowers that were inoculated by hand or previously visited by infected bumble bees [11]. More recently, the parasites *Apicysistis bombi*, *Nosema spp*., and *Crithidia bombi* were vectored from host bees to flowers and between bee species through shared flowers [10,13]. Though this work shows that flowers can act as bridges for pathogens moving between species and/or populations, the mode of transmission for the multitude of RNA viruses that infect bees is still unknown.

Once thought to be specific to honey bees, RNA viruses have been detected in a number of pollinating arthropod species including beetles, flies, solitary bees, and bumble bees [6,9,14]. These single stranded positive sense viruses are highly prevalent among honey bees and usually persist as covert infections capable of replicating under certain conditions, such as pesticide exposure and immunosuppression induced by *Varroa* mites, an ectoparasite that vectors RNA viruses [15,16]. Two of the most common in the United States are deformed wing virus (DWV), which causes wing deformities in both honey bees [17,18] and bumble bees [19], and black queen cell virus (BQCV) which causes the blackening and deadening of queen cells in honey bees [20]; however the effects on bumble bees are unknown. In honey bees, *Varroa* mites serve as a vector for RNA viruses with high infestations typically resulting to high virus titers.

Managed bees including both honey bees and commercial bumble bees have been implicated in the spread of numerous pathogens and parasites [21–25]. Evidence suggests that RNA viruses are also spilling over from managed honey bees into wild bees. In the United Kingdom (UK), sympatric bees share the same virus strains [23] and virus prevalence in honey bees is a significant predictor of virus prevalence in wild bumble bees [25]. Recent research suggests that managed honey bee apiaries may be hotspots for RNA viruses. Bumble bees are more likely to host viruses when collected near honey bee apiaries and only flowers collected near apiaries were found to harbor bee viruses [12]. In addition, the global spread of DWV is tightly linked to the movement of *Apis mellifera* and the *Varroa* mite [26]. However, other bee species are not known hosts of the *Varroa* mite. Additional studies are needed to closely examine the principle directionality of transmission as well as transmission routes of viruses among species.

Detected in the feces and glandular secretions of worker honey bees as well as in pollen loads carried by bees, RNA viruses are likely left behind on flowers by foraging visitors [9,27,28]. Previous work has also shown that virus particles on pollen grains can remain infective for six months in ambient conditions [9]. Thus, the suggestion has been made that flowers serve as platforms for RNA virus spread to visiting arthropods. However, to our knowledge, only one previous study has tested the transmission of RNA viruses between bee species as a result of using flowers. In a controlled flight cage experiment, Israeli acute paralysis virus (IAPV) was transmitted between honey bee and bumble bee colonies that foraged alongside each other for several weeks. Although shared flowers may have provided the transmission route, bees might also have become infected by direct contact either by comingling or if bees entered each other’s hives through resource robbing or drifting [9]. In addition, it is not yet known whether transmission occurs through single or chronic exposure to contaminated flowers. Although Singh et al. (2010) were instrumental in demonstrating transmission between bee species, the role of flowers in RNA virus transmission remains unclear.

Transmission via flowers may be mediated by many factors such as plant traits and/or pollinator behavior. For example, the ability for flowers to serve as conduits for pathogens may be facilitated or constrained by floral traits or morphology [5,29]. In the previous studies examining parasite transmission through flowers, parasites were unequally dispersed across plant morphotypes [11] and species [10,29] suggesting that floral architecture may influence dispersal and transmission rate. Floral architecture may influence visit duration and how a bee physically contacts a flower [30], which may impact the likelihood of virus deposition or acquisition. A plants’ propensity to harbor viruses could also be related to pollinator visitation rates with highly attractive plants more likely to act as fomites. Floral traits such as floral size, corolla depth [31], color [32], as well as nectar production rate and composition [33,34] may all influence pollinator visitation rates. Flowering plant diversity may also be an important factor as resource availability may impact floral preference of foraging bees [35,36]. More research is needed to fill these knowledge gaps in viral transmission [5,37].

Here, we conducted a series of controlled flight cage experiments to test if flowers can act as bridges in virus transmission between bee species. Specifically, we examined if honey bees deposit viruses on flowers and whether bumble bees become infected after visiting contaminated flowers. In addition, we examined whether virus deposition is influenced by plant species and/or plant diversity. By measuring visitation to flowers, we examined whether honey bee visitation rates and/or visit duration increase the likelihood of virus deposition on flowers. To further examine the role of flowers in the transmission of RNA viruses, we tested whether virus transmission from honey bees to bumble bees occurs indirectly through flowers, including chronic exposure to contaminated flowers. We also tested whether virus transmission occurs through direct contact or comingling while foraging on flowers.

## Material and methods

### Experimental overview

**Fig 1:**
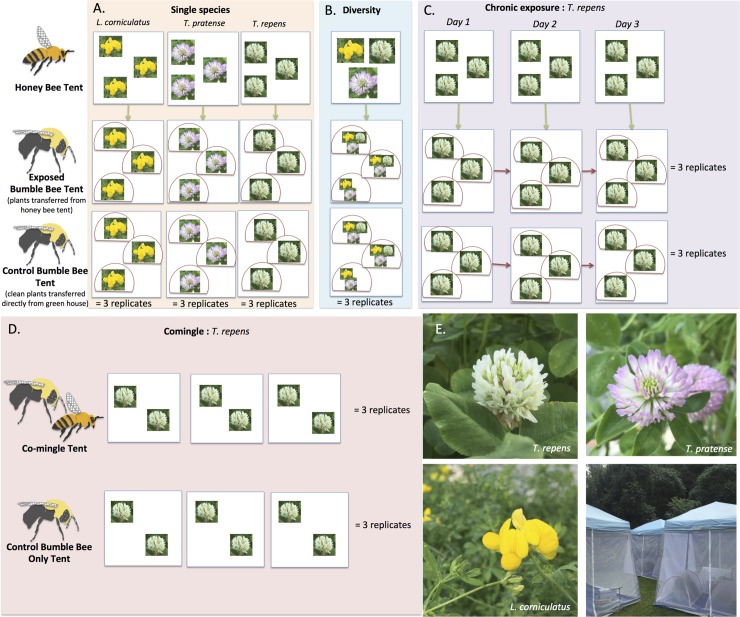
Schematic of experimental designs. In a series of four experiments, we examined virus deposition on flowers by honey bees and/or virus transmission between honey bees and bumble bees. To examine the effect of plant species and/or plant diversity, flowering plant species were provided to foraging bees as either single plant species (A) or in diverse arrays consisting of all three species (B). To test whether chronic exposure to contaminated flowers is necessary for virus transmission, bumble bee microcolonies were exposed three times to honey bee-visited flowers over the course of three days (C). To test if direct contact or comingling is necessary for viral transmission, honey bees and bumble bees were allowed to forage together in tent enclosures (D). Blue boxes in the schematic represent tent enclosures assigned as the honey bee tent (where infected honey bees were allowed to forage on flowers), the exposed bumble bee tent (where plants exposed to honey bees were transported into three hoop houses to be foraged on by bumble bee microcolonies), and the control bumble bee tent (where bumble bee microcolonies foraged on ‘clean’ plants brought directly from the greenhouse). Red semi-circles represent hoop houses within bumble bee tents, each containing a single bumble bee microcolony. Green arrows represent the movement of plants from the honey bee tent to the exposed bumble bee tent after a 15 hour nectar regeneration period. In the chronic experiment, the same three bumble bee colonies were used on each of three days (depicted by red arrows connecting the hoop house through time). Three plant species were used throughout the series of experiments: *Trifolium repens*, *T*. *pratense*, and *Lotus corniculatus*. Photos of inflorescences and tent enclosures (with hoop houses) are provided for visualization (E).

To test for viral deposition on flowers by honey bees and transmission of viruses between bee species using shared floral resources, we conducted a series of experiments (Fig 1). First, we allowed infected honey bees to forage on arrays of flowering plants within a screened enclosure and later transferred these plants to enclosures where non-infected bumble bees were allowed to forage. We tested all bees and flowers after each experiment. All foraging trials were conducted in Burlington, Vermont (44°29'07.2"N 73°11'12.6"W).

### Setup and pre-screening

To focus on floral architecture and reduce variation in other plant traits, we chose three legume species (Family Fabaceae), two of which having similar morphology: *Trifolium pratense* (red clover), *Trifolium repens* (white clover) and *Lotus corniculatus* (birdsfoot trefoil) (Fig 1E). The two *Trifolium* species are similar but differ from *Lotus corniculatus* in terms of floret number and inflorescence shape, traits that could influence bee visitation rates, visit duration, and how bees contact the floral surface, and lead to differences in viral deposition to floral surfaces. The inflorescences of *T*. *pratense* and *T*. *repens* are terminally borne on stems and consist of globulose clusters of 20–40 tubular flowers. The inflorescences of *L*. *corniculatus* are umbel-like cymes, consisting of eight flowers, borne at the end of axillary branches. We chose three plant species that we found to be highly visited in the field by both honey bees and wild bumble bees (unpublished data) and widely grown in agriculture as nitrogen fixing cover crops and fodder. We grew plants from seed and maintained them in a greenhouse until the start of the experiment. Beginning in mid-May, we broadcast seeds (Seedway, NY) of *T*. *pratense*, *T*. *repens*, and *L*. *corniculatus* in 8 in. diameter, 6.5 in. deep plastic pots filled with Miracle Grow Potting Mix to achieve ca. 100 seeds per pot. To encourage flowering, we trimmed the *T*. *repens* and *T*. *pratense* plants once and twice, respectively, and used grow lights to maintain 14 hours of sunlight. To verify that plants were virus-free at the start of the experiment, we haphazardly collected three composite samples of 1.5 grams from each plant species and tested them for DWV and BQCV using RT-qPCR protocols.

To ensure our experimental honey bee colonies were infected with both viruses, we collected 50 bees from each of two five-frame honey bee colonies (University of Vermont research colonies), and tested each composite sample for DWV and BQCV using RT-qPCR. Thus, we knew that these colonies were infected with both viruses. We received seven bumble bee colonies from a commercial supplier (BioBest). To verify these bees were not infected with DWV and BQCV, we pollen-starved 10 bees from each colony for 72 hours and tested each bee using RT-qPCR. Bees were pollen-starved to rid their guts of pollen that may have contained virus particles. All bumble bee colonies tested negative for DWV and BQCV. From the seven colonies, we created microcolonies of 12 adult bees, provided them with 30% sucrose solution *ad libitum* and allowed them to acclimate for up to five days in a growth chamber maintained at 26°C and 52–55% RH. We made new microcolonies every three days to ensure each microcolonies used in the experiment was approximately the same age.

We carried out all experiments in three 3 x 3 x 3 m. screened tents with tarp bottoms. Each tent was assigned to one treatment: honey bee tent, control bumble bee tent, or exposed bumble bee tent (Fig 1E). We used one additional tent as a plant holding area to keep unwanted insects from visiting the plants during the experiment. To restrict bumble bees to a smaller foraging area, we set up three hoop houses within each of the two bumble bee control and exposed tents. Hoop houses (1 x 1 x 0.7 m) were constructed of white fabric stretched and stapled over two pieces of arching PVC tubes that were screwed to a wooden frame.

### Experimental design

On each day of the experiment, we transported plants from the greenhouse to the plant holding tent and watered them. We counted all inflorescences to ensure a standard range across replicates and treatments and assigned them accordingly. To acclimate the honey bees to their enclosure, the two colonies (consisting of five frames each) were placed in the honey bee tent 24 hours prior to the experiment. To infect the flowering plants, we placed plants within the screened enclosure with the two honey bee colonies and allowed bees to visit the flowers. After the foraging trials, we transferred plants to a holding tent to allow for nectar to be replenished. After 15 hours, we transferred plants visited by honey bees to the exposed bumble bee tent and evenly distributed them among the three hoop houses each containing a single bumble bee microcolony for a total of three replicates per experiment. At the same time, we ran three replicate controls for each experiment in three hoops houses within the control bumble bee tent. Bumble bees in the control tent received clean flowering plants brought directly from the greenhouse. All trials were conducted in the shade to reduce UV exposure to flowers and degradation of viral RNA. On each day of foraging trials, honey bees and bumble bees were allowed to forage on floral arrays for nine and six hours, respectively. For the comingle experiment, where both honey bees and bumble bees were allowed to forage together in the same tent, bees were allowed to forage for seven hours. These foraging times were chosen to maximize the amount of foraging time allowed in a single day to increase the probability of detecting an effect.

To measure visitation, we observed bumble bee foragers until 50% of flowers for each replicate were visited. To closely examine how honey bee visitation may influence virus deposition on flowers, we filmed each trial for three hours. We viewed the videos and recorded the total number of honey bee visits to each plant species and computed the visit duration of each foraging honey bee (in seconds) to each inflorescence during the filmed visitation surveys.

For each trial, we allowed microcolonies of 12 bees each to forage on flowers that had or had not been exposed to honey bees. After six hours, we collected all inflorescences and bumble bees. We stored inflorescences at -80°C. We placed the bumble bee microcolonies into new containers and fed 30% sucrose *ad libitum* for one week in a growth chamber. If bumble bees were exposed to infective virus during the experiment, the one week ‘incubation’ period allowed for the onset of viral infection. We did not feed bees pollen during this period to clear their guts of pollen that could contain inactive virus particles, resulting in a false positive result during the viral assays. After one week, we collected all bees and stored them in -80°C until RNA extraction and virus assays.

To test if plant species influences virus deposition and/or the transmission of virus between bee species, we conducted the above-described foraging trials with three replicates for each plant species: *T*. *repens*, *T*. *pratense*, and *L*. *corniculatus* (“single species” experiment) (Fig 1A). We standardized the number of inflorescences used in each replicate: 15–20 *T*. *repens* inflorescences, 13–15 *T*. *pratense* inflorescences, and 31–40 *L*. *corniculatus* inflorescences. Because *L*. *corniculatus* inflorescences contain less than half the number of florets as *T*. *pratense* and *T*. *repens*, we used approximately twice as many inflorescences.

To test if plant diversity affects virus deposition and/or transmission, we allowed bees to forage on floral arrays containing all three plant species at once (“diversity” experiment) (Fig 1B). Each diversity array was replicated three times and each consisted of 7–8 *T*. *repens* inflorescences, 6 *T*. *pratense* inflorescences, and 15–21 *L*. *corniculatus* inflorescences. For both the single plant species and diversity trials, we collected separate samples of each plant species after each replicate for a total of three flower samples per species per trial for each of the exposed and control groups.

To test if chronic exposure to contaminated flowers is necessary for virus transmission, we repeated the experiment using *T*. *repens* (“chronic exposure” experiment) for a total of three replicates (Fig 1C). Six bumble bee microcolonies were either assigned the exposed treatment group or control group and allowed to forage on exposed or unexposed *T*. *repens* plants on three consecutive days (six hours each day). We allowed plants to replenish nectar between honey bee and bumble bee foraging bouts as in the other experiments. A new *T*. *repens* plant was used each day. After the three exposure events, we collected all bumble bees, transferred them to new containers, provided 30% sucrose ad libitum, and ‘incubated’ them for one week as in the previous experiments and then transferred them at -80°C. We also collected flowers each day of the chronic exposure experiment and stored them at -80°C. Since new plants were used each day, we collected a total of nine exposed flower samples and nine control flower samples.

To test if direct exposure, or comingling, on flowers is necessary for transmission of viruses between bee species, we used bumble bee colonies comprised of 75–100 workers and *T*. *repens* arrays consisting of 41–47 inflorescences (“comingling” experiment) (Fig 1D). We placed two honey bee colonies, a single bumble bee colony, and pots of *T*. *repens* plants into a tent enclosure. For the control, we placed a single bumble bee colony with plants into a separate tent enclosure. We allowed all bees to forage on the plants for a total of seven hours, during which we observed until both honey bees and bumble bees visited over 50% of the flowers present for each replicate. After seven hours, we returned all foraging bumble bees back to their colony box and transferred them back to the growth chamber. This was repeated three times over the course of three days using the same honey bee colonies but different bumble bee colonies. We fed the bumble bee colonies pollen and 30% sucrose *ad libitum* for three weeks in growth chambers to encourage the spread of viruses throughout the colony. After three weeks, we made pollen-starved microcolonies consisting of 12 bees. After a one-week pollen starvation period, we collected these bees and stored them at -80°C. Two flower samples were collected from each replicate for a total of six exposed flower samples and six control flower samples.

### RNA extraction

We extracted total RNA following Qiagen RNeasy mini kit protocols. The entire abdomen of each individual bumble bee was dissected and flash frozen on N_2_ and homogenized into 600 ul of RLT buffer (10% β-mercaptoethanol) and Qiagen protocols were used thereafter for each individual bumble bee. For the pre-screening of honey bees, samples of 50 bees were pooled, flash frozen in N_2_ and homogenized together in an extraction bag with 10 mL of GITC buffer. The resulting homogenate was centrifuged and 100 ul of the lysate was mixed with RLT buffer (10% β-mercaptoethanol) and Qiagen protocols were used thereafter. For both pre-screened and experimental plants, 1.5 g of flower material consisting of entire inflorescences was transferred to an extraction bag (Bioreba, Switzerland) and flash frozen in N_2_. Plant material was ground to a powder using a ceramic pestle on the outside of the extraction bag for 30 seconds. Three mL of GITC buffer was added to the bag and the pestle again was used on the outside of the bag to mix the homogenate into the buffer for 2 minutes. The resulting homogenate was centrifuged and 200 ul was used in RNA extractions following Qiagen RNeasy mini kit protocols. All RNA quantity and quality were assessed on a Spectrometer (Nanodrop, Thermo Scientific).

### Virus detection and quantification

For bumble bees and honey bees, all RNA extractions were diluted to 20 ng/ul prior to virus assays. RNA recovered from plants was not diluted prior to further analyses. For reverse transcription of RNA and absolute quantification, duplicate reverse transcription quantitative polymerase chain reaction (RT-qPCR) was performed for each sample with SYBR green one-step RT-qPCR kit in 10 ul reactions using the following thermal cycling program: 10 min at 50°C (RT) followed by 1 min at 95°C, and 40 amplification cycles of 95°C for 15 s, 60°C for 60s. Last, the melt-curve was obtained starting at 65-95°C (0.5°C increments, each 2 s). We used primers specific to the positive strand of the following RNA virus targets: DWV and BQCV, and a housekeeping gene (ACTIN) as a positive control of RNA extraction efficiency (S1 Table). Quantification was calculated using duplicate standard curves of gBlocks Gene Fragments that were developed using double-stranded, sequence verified genomic blocks consisting of the four targets of interest separated by ten random base pairs (S1 Supplemental Information). Sequences of random base pairs consisting of at least 50% G and Cs were used at the beginning and terminal ends of the fragment. Efficiencies were 91.06% (DWV), 95.21% (BQCV), and 90.12% (Actin), with correlation coefficients (R^2^) ranging from 0.993–0.999.

### Sequencing

To confirm the identity of the viruses, we sequenced virus fragments from bumble bees, honey bees and flowers. qPCR product was cleaned (ExoSAP-IT PCR Product Cleanup) and sequencing was performed using the 3130xl Genetic Analyzer in the University of Vermont Cancer Center Advanced Genome Technologies Core. Sequence data were viewed for quality assessment (FinchTV 1.4) and aligned by eye to genome references using Geneious v 6.0.6 (BQCV: GenBank: KY243932.1; DWV: GenBank: KJ437447.1).

### Data reporting and analysis

We refer to virus prevalence on flowers as the percentage of flower samples with virus in each experimental trial. Virus loads are presented as the number of genome copies per flower sample or bee. Visitation rate was calculated as the total number of honey bee visits per hour per plant species. Visit duration was measured as the amount of time honey bee foragers were observed visiting inflorescences (in seconds). Since plants in the chronic exposure trials were experimentally treated the same as plants in the single species trials, we combined these data together in analyses examining virus deposition on plants. Data from the comingle trials were omitted from analyses of virus deposition on plants, as these trials were only conducted to examine transmission via direct contact between bee species and had different experimental conditions that could confound results (number of inflorescences, foraging time)

To examine the effect of plant species, virus species, and their interaction on virus prevalence (here analyzed as presence/absence) on flowers used in single species trials, we used a generalized linear mixed effects model (GLMM) with flower sample as a random effect. In order to examine prevalence of RNA viruses on flowers, the model was structured such that each flower sample was included twice, once for each virus. To examine the interaction effect of plant diversity and plant species on virus prevalence, we conducted a separate GLMM testing the interaction of diversity (single species vs. mix of three species) and plant species with flower sample as a random effect. Since visitation rate and visit duration could additively affect virus deposition to flowers, we conducted a third GLMM including visitation rate and visit duration as fixed effects and included flower sample as a random effect. All GLMM models on virus prevalence were conducted with a binomial distribution (link = “logit”). Virus loads of contaminated flowers were log_10_ transformed to achieve normality prior to analyses. For virus load, we conducted linear mixed models (LMM) with identical structures, terms, and random effects as the GLMMs. The interaction of diversity (single species vs. mix of threes species) and plant species was not included in virus load model as the model was rank deficient and unable to compute the interaction term [38]. As no bumble bees were infected in the trials, we could not test the effect of the single species, diversity, chronic, or comingling experiments on the prevalence or virus load in bumble bees.

All mixed effects models were conducted using the LME4 package using the glmer() function for virus prevalence and the lmer() function for virus load [39]. Significance for all models was determined by comparing full and reduced models with likelihood ratio tests. We examined pairwise comparisons using Tukey contrasts in the MULTCOMP package, using the glht() and mcp() functions [40]. To avoid errors associated with post hoc tests on interacting variables, pairwise comparisons were conducted only on significant main effects with three or more factor levels in models with non-significant interaction effects. We conducted all analyses using the statistical software “R” v 3.5.1 [41].

## Results

At the onset of the experiment, all plant species were negative for DWV and BQCV. RNA virus loads in the two honey bee colonies were 10^4^ and 10^9^ genome copies per bee for DWV and 10^8^ and 10^6^ genome copies per bee for BQCV. All bumble bees were negative for both viruses at the onset of the experiment (n = 70). No bumble bees became infected in any of the experiments (single plant species, diversity, chronic exposure, comingling) for either the control group (n = 192) or exposed group (n = 220).

All flowers visited only by bumble bees (control groups) were negative for both viruses. Of the flowers visited by both honey bees and bumble bees, we detected DWV and BQCV on 24.2% and 21.2%. When single species of plants were offered to infected honey bees, we detected viruses on all three species (Table 1). However, we found a significant interaction effect of plant species and virus species (χ_2_^2^ = 11.15, p = 0.004), such that DWV and BQCV were not equally distributed across plant species (Table 2). Main effects of plant species and virus species were not significant. In the diversity trials, where all three plant species were offered together, we only detected viruses on *T*. *pratense* (Table 1). By analyzing data sets from the single plant and diversity experiments, we found a significant interaction of plant species and experiment (χ _2_^2^ = 17.91, p < 0.001; Table 2). Flowers that received the longest visits by honey bee foragers were more likely to be contaminated with viruses (χ _2_^2^ = 4.076, p = 0.044, Table 2). However, we saw an opposite trend with visitation rate. Flowers that received the fewest number of visits were less likely to be contaminated with viruses (χ _2_^2^ = 5.452, p = 0.020, Table 2).

**Table 1:** Summary table showing the prevalence of deformed wing virus (DWV) and black queen cell virus (BQCV) on three plant species across all foraging trials where both honey bees and bumble bees foraged. Plants foraged by bumble bees only were all negative for viruses and are therefore excluded from this table. Virus prevalence is reported as the number of flower samples with virus detected divided by the total number of flower samples tested for each trial (n) multiplied by 100. Total column provides the virus prevalence for each plant species across all experiments.

Virus	Plant species	Virus Prevalence (n)				
		Single species	Diversity	Chronic	Comingle	Total
**DWV**	*L*. *corniculatus*	0 (3)	0 (3)	-	-	0 (6)
	*T*. *pratense*	33.3 (3)	100 (3)	-	-	66.6 (6)
	*T*. *repens*	66.6 (3)	0 (3)	11.1 (9)	16.6 (6)	20 (21)
**BQCV**	*L*. *corniculatus*	100 (3)	0 (3)	-	-	50 (6)
	*T*. *pratense*	0 (3)	100 (3)	-	-	50 (6)
	*T*. *repens*	0 (3)	0 (3)	11.1 (9)	0 (6)	5 (21)

**Table 2:** Summary statistics for all statistical models. For each model, the response variable and predictor variables are outlined with relevant summary of statistics. Virus prev. is virus prevalence and is reported as the number of plants samples with virus detected divided by the total number of plant samples in the dataset multiplied by 100. Virus load is presented as virus genome copies per flower sample. Plant spp. refers to the plant species used in the experiments: *Lotus corniculatus* (Birdsfoot trefoil), *Trifolium pratense* (red clover), or *Trifolium repens* (white clover). Virus species are either deformed wing virus (DWV) or black queen cell virus (BQCV). Diversity is either ‘single species.’, ‘mix of three species’. In the “single species” experiment, bees foraged on arrays consisting of only one species at a time. In the “mix of species” experiment, bees foraged on arrays consisting of all three plant species at once. Visitation (visitation rate) was calculated as the number of honey bee visits to flowers/hour. Duration (visit duration) was calculated as the amount of time each honey bee forager visited to an inflorescence (in seconds).

Response variable *Model*:	Predictor variable(s)	Test	Family	Test stat.	df	P	Sig.^a^
**Virus prev.**				*χ*^2^			
*Virus species*:	Plant spp.	GLMM	binom.	2.787	2	0.248	ns
	Virus spp.	-	-	0.00	1	1.00	ns
	Virus spp. : Plant spp.	-	-	11.15	2	0.004	**
*Diversity*:	Plant spp.	GLMM	binom.	6.996	2	0.030	*
	Diversity	-	-	0.006	1	0.939	ns
	Diversity : Plant spp.	-	-	17.91	2	0.001	***
*Visits/Duration*:	Visitation	GLMM	binom.	5.452	1	0.020	*
	Duration	-	-	4.076	1	0.044	*
**Virus load**				*χ*^2^			
*Virus species*:	Plant spp.	LMM	Gaus.	18.03	2	0.001	***
	Virus spp.	-	-	2.367	1	0.124	ns
	Virus spp. : Plant spp.	-	-	0.667	1	0.414	ns
*Diversity*:	Plant Spp.	LMM	Gaus.	23.70	2	0.001	***
	Diversity	-	-	9.968	1	0.002	**
*Visits/Duration*:	Visitation	LMM	Gaus.	5.174	1	0.023	*
	Duration	-	-	2.223	1	0.136	ns

^a^Asterisks represent level of significance

* P < 0.05

** P < 0.01

***P < 0.001

**Fig 2:**
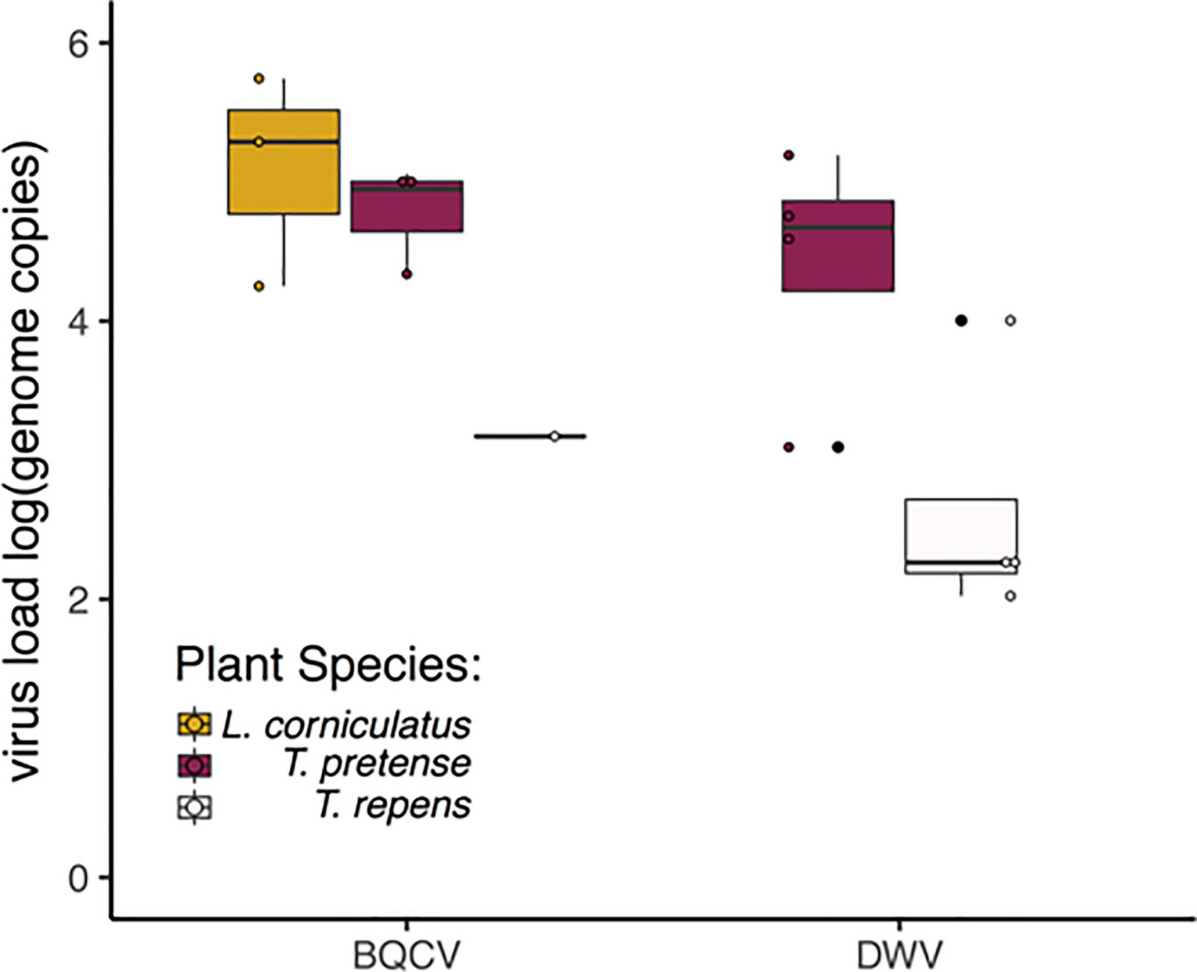
Virus load for virus positive flower samples by plant species across all trials. Box plots color coded by plant species. Whiskers represent max and min, the box edges are the 1st and 3rd quartiles and the middle line represents the median. Deformed wing virus (DWV), black queen cell virus (BQCV). Plant species are *Lotus corniculatus* (birdsfoot trefoil), *Trifolium pratense* (red clover), or *Trifolium repens* (white clover).

Virus loads on flowers ranged from 10^3^−10^5^ genome copies (Fig 2). In single species diversity trials, virus loads differed across plant species (χ _1_^2^ = 18.03, p < 0.001, Table 2) and were lowest on *T*. *repens* compared to *T*. *pratense* (p = 0.02) and *L*. *corniculatus* (p = 0.005) but did not differ by virus species (χ _2_^2^ = 2.367, p = 0.124). The interaction of plant species and virus species was not significant for virus load. Virus loads were different across diversity (single plant species vs. mix of three species) with the highest virus loads occurring on plants in the mix of three species trials (χ _1_^2^ = 9.968, p = 0.002). Flowers that received fewer honey bee visits had the highest virus loads (χ _2_^2^ = 5.174, p = 0.023). Visit duration did not influence virus loads left behind on flowers by honey bee foragers (χ _2_^2^ = 2.223, p = 0.136).

## Discussion

Although flowers have been implicated as bridges in the spread of bee diseases [5], the role of flowers in the transmission of RNA viruses among pollinator species has remained largely unstudied. Using a series of foraging experiments with captive honey bee colonies and arrays of flowering plant species, we experimentally demonstrated that honey bees deposit viruses on flowers. We also found evidence that flowering plant species and/or bee behavior may influence the likelihood of virus deposition. Our study is among the first to closely examine the role of flowers in bee virus transmission and is the first to demonstrate virus deposition on flowers by honey bees.

Deformed wing virus and BQCV were differentially deposited across the three plant species, indicating that modes of deposition vary for virus species and that deposition may be mediated by floral traits. In our study, virus deposition on *L*. *corniculatus* was unique in that BQCV was detected in all samples yet no DWV was detected. In contrast, DWV and BQCV were detected on both *Trifolium* species during the course of the study. Compared to the *Trifolium* species, the inflorescences of *L*. *corniculatus* are the least similar morphologically and consist of only eight florets (compared to 20–40 in *Trifolium* species). To visit the nectaries of *Trifolium*, pollinators access nectaries by probing multiple tubular flowers while crawling about the surface of the inflorescence head. For *L*. *corniculatus*, nectaries may be accessed through contact with only the anterior portion of the bee. If a virus is deposited by feces, floral morphology that encourages ‘hovering’ behavior, may reduce the likelihood of viral deposition [5]. Likewise, for viruses deposited through oral secretions, complex inflorescences with numerous florets to probe may increase the likelihood of viral deposition. In other bee-pathogen systems, plant traits such as floral morphology [10,11] or number of open flowers [29] are identified as important factors influencing transmission. To understand the specific floral traits that mediate virus deposition, future studies should take quantitative measurements on floral attributes and manipulate traits such as number of florets and corolla depth. Singh et al. (2010) noted that virus species detected in honey bees and their corresponding pollen loads differed considerably; suggesting differences in viral ecology, and/or differences in pollinator contact with contaminated pollen [9]. Investigating differences in how different RNA viruses are shed from visiting pollinations would also help to explain the interaction effect.

We found flowers that received longer visits by honey bees were more likely to host viruses. However, flowers with higher visitation rates were less likely to host viruses and also had lower virus loads. These results underline the complexity of this study system and the need to understand how viruses are shed from bees onto flowers during foraging bouts.

We saw an interaction of floral diversity and plant species that is not explained by differences in visit duration alone. When bees foraged on single-species floral arrays, viruses were deposited on all three species. However, when bees were offered diverse arrays consisting of all three plant species, we only detected viruses on *T*. *pratense*, despite no difference in visit duration for *T*. *pratense* between diversity trials (single species vs. mix of three species) (S1 Fig). Our results could be explained if honey bee colonies hosted both infected and uninfected individuals that foraged differently as a result of infection status. Foraging differs for parasite infected bees than for those that are uninfected, suggesting that bees seek benefits from the medicinal properties of secondary plant metabolites [42–46]. Compared to *T*. *repens*, *T*. *pratense* has substantially higher concentrations of isoflavonoids [47], a group of phenolic compounds that possess antiviral properties against a wide range of viruses [48]. However, we were unable to distinguish between infected and uninfected bees at the outset of the experiment. Future work should examine potential differences in foraging behavior of individuals infected with RNA viruses.

Under our experimental conditions, bumble bees did not develop an infection after direct contact with honey bees through comingling or indirect contact through shared flowers. These results indicate that transmission of viruses between bee species through flowers is a rare occurrence, with experimental detection contingent on numerous factors. For example, factors such as immunocompetence, virus virulence, virus load, and the probability a bumble bee will contact a virus particle on a flower may all contribute to detection. Though, transmission through flowers may have a low probability, the high prevalence of contaminated flowers and high flower visitation rates exhibited by bees in the wild may be hallmarks of a process that occurs with frequency in nature but is difficult to capture in an experimental setting. We also note that we did not test whether the virus strains in our honey bees were infective to bumble bees. Thus, although we did not demonstrate virus transmission to bumble bees in our experiment, we remain cautious to exclude the possibility under different experimental conditions and with greater sample sizes.

Our findings present several promising avenues for future research. We were successful in demonstrating virus deposition to flowers by honey bees under experimental conditions. To test whether our results are relevant in nature, future studies should test field-collected flowers near honey bee apiaries. Since other bee species may also deposit viruses on floral resources, selecting field sites with varying densities of honey bees and measuring floral visitation could shed light on the importance of honey bees versus other bees to virus deposition on flowers. To further understand directionality of transmission, future experiments should test whether infected bumble bees and/or other bee species will also deposit viruses on flowers [49]. In addition, future experiments should focus on the second half of the transmission route and examine whether bumble bees and/or other bee species can acquire virus particles or become infected after visiting inoculated flowers. Our results suggest that flowering plant species may differ in their propensity to harbor viruses. Thus, closely examining the mechanisms of virus deposition in conjunction with floral traits could help to explain the differences we observed. Lastly, additional behavioral studies are needed to examine how foraging behavior may be affected by viral infection.

## Supporting information

S1 Table Primers used for the amplification of RNA virus and actin amplicons. (PDF)

S1 Supplemental Information gBlocks Gene Fragments (Integrated DNA Technologies) sequence. Virus and actin amplicons are colored for visualizations: Green = DWV, Blue = IAPV, Red = Actin, Yellow = BQCV. Ten random base pairs (uncolored) flank each target of interest.
(PDF)

S1 Fig Honey bee visit duration and visitation rate by plant species and experiment. Box plots show median duration of honey bee visits to three plant species: *Lotus corniculatus* (birdsfoot trefoil), *Trifolium pratense* (red clover) and *T*. *repens* (white clover). Colors of box plots represent data from the “diversity” experiment, where all plant species were provided at the same time, and “single species” where each plant species were provided individually. Letters above box plots show results of pairwise comparisons for visit duration data. Red lines show the visitation rate (number of honey bee visits/hour) to each plant species for each experiment. Visit duration data were log_10_ transformed to achieve normality prior to analysis. We examined the effect of plant species on visit duration in an ANOVA using data from the single species experiment trials. We examined pairwise comparisons using Tukey contrasts (R library multcomp, functions glht and mcp). In a separate ANOVA using data from the single species and diversity trials, we examined the interaction effect of plant species and experiment (single species vs. diversity) on visit duration.
(TIF)
