# Correction: Flowers as viral hot spots: Honey bees (*Apis mellifera*) unevenly deposit viruses across plant species

**DOI:** 10.1371/journal.pone.0225295

**Published:** 2019-11-11

**Authors:** The PLOS ONE Staff

## Notice of republication

This article was republished on October 30, 2019, to replace a corrupted version of S1 Fig. The publisher apologizes for this error. Please download this article again to view the correct version.
