# Supplementary material for: Flowers as viral hot spots: Honey bees (Apis mellifera) unevenly deposit viruses across plant species
Source: PLoS One. 2019 Sep 18;14(9):e0221800. doi: 10.1371/journal.pone.0221800 (PMC6750573; doi:10.1371/journal.pone.0221800)
Supplement: S1 Table — (PDF) [file pone.0221800.s001.pdf]

S1 Table.

Primer	5' to 3' Sequence	Product Size (bp)	Annealing Temp (^oC)	Reference
DWV-F	TTCATTAAAGCCACCTGGAACATC	136	53	(Traynor et al., 2016)
DWV-R	TTTCCTCATTAACTGTGTCGTTGA			
BQCV-F	TTTAGAGCGAATTCGGAAACA	140	51	(Traynor et al., 2016)
BQCV-R	GGCGTACCGATAAAGATGGA			
Actin-F	CGTGCCGATAGTATTCTTGC	138	56	
Actin-R	CCATTGTCAACTACGAGTGC			
