# Supplementary material for: Flowers as viral hot spots: Honey bees (Apis mellifera) unevenly deposit viruses across plant species
Source: PLoS One. 2019 Sep 18;14(9):e0221800. doi: 10.1371/journal.pone.0221800 (PMC6750573; doi:10.1371/journal.pone.0221800)
Supplement: S1 Supplemental Information — Virus and actin amplicons are colored for visualizations: Green = DWV, Blue = IAPV, Red = Actin, Yellow = BQCV. Ten random base pairs (uncolored) flank each target of interest. (PDF) [file pone.0221800.s002.pdf]

S1. Supplemental Information

GGACGGACAGTTCATTAAAGCCACCTGGAACATCAGGTAAGCGATGGTTGTT
TGACATTGAGCTACAAGACTCGGGATGTTATCTCTTGCGTGGAATGCGTCCCG
AACTTGAGATTCAATTATCAACGACACAGTTAATGAGGAAAACCATGTACGC
CATGCCTGGCGATTCACAACAAGAAAGCAATACTCCCAATGTACACAACACG
GAACTCGCTTCGTCAACTAGTGAAAACTCGGTTGAGACCCAAGAAATCACAA
CCTTTCATGATGTGGAAACTCCAAATAGGATCGATACCCCCATGGCTCAGGA
TACTTCATCGGCTAGGAACATGGATGATACGCACAGTATTATTCAGCTTCCCT
GCTCGTGCCGATAGTATTCTTGCGGTGTCTCTTTGCCGATCAACGATCGTGTA
CTTTGTTGGTTACCTTCGATTCTAAAAGATAACTCAATAAACCAAACATGTGT
GACGAAGAAGTTGCTGCACTCGTAGTTGACAATGGCGTCCACCTGTTTAGAG
CGAATTCGGAAACATTTTACTATAGTTCAGGTCGGAATAATCTCGATATAGCC
ACTTCACCTCCTTCCATCAATCGCTACTATGCGGTAGGTGCGGGAGATGATAT
GGACTTTTCCATCTTTATCGGTACGCC ATGAGCGCCA
