# Supplementary figures and images for: Flowers as viral hot spots: Honey bees (Apis mellifera) unevenly deposit viruses across plant species
Source: PLoS One. 2019 Sep 18;14(9):e0221800. doi: 10.1371/journal.pone.0221800 (PMC6750573; doi:10.1371/journal.pone.0221800)

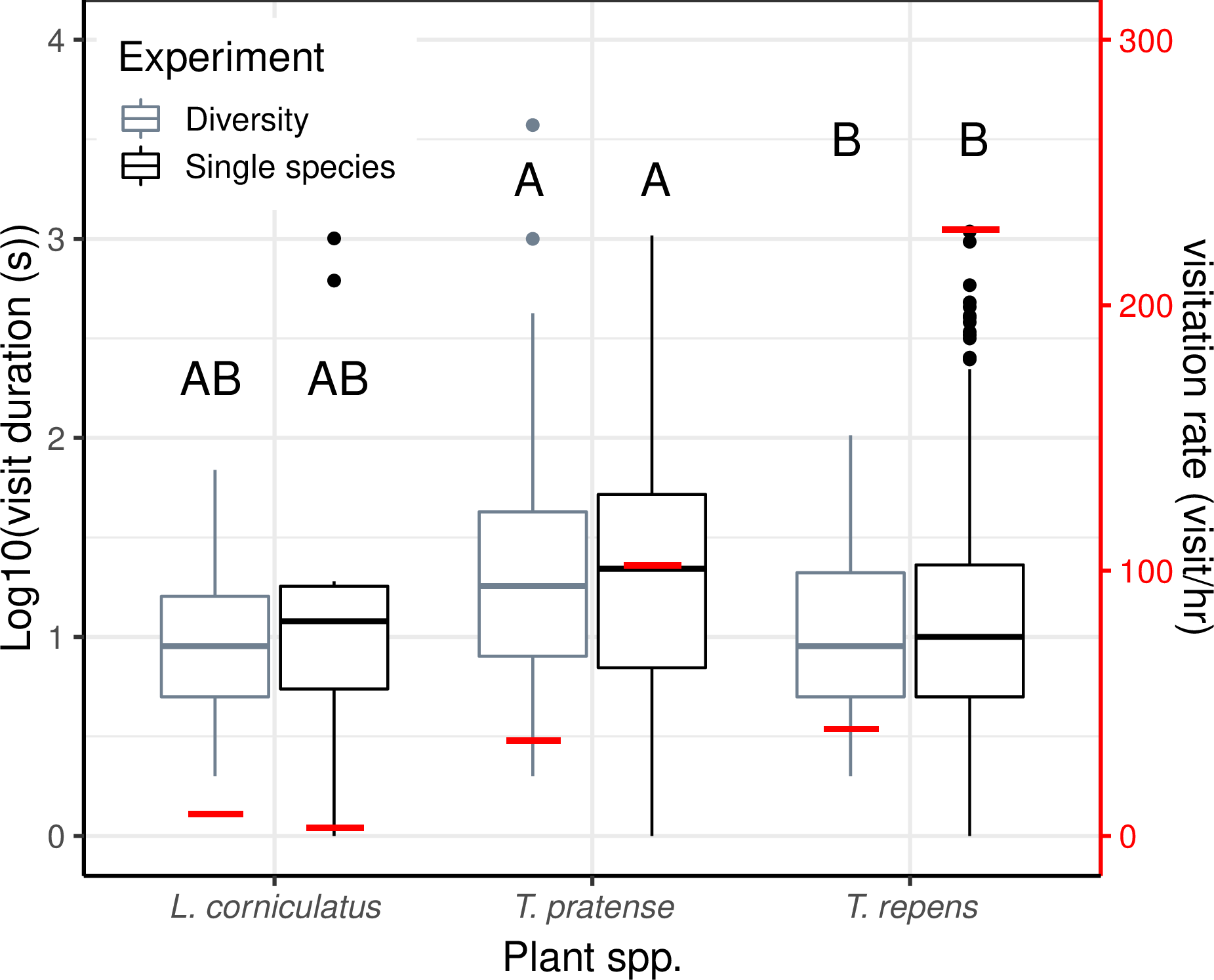

Supplement: S1 Fig — Box plots show median duration of honey bee visits to three plant species: Lotus corniculatus (birdsfoot trefoil), Trifolium pratense (red clover) and T. repens (white clover). Colors of box plots represent data from the “diversity” experiment, where all plant species were provided at the same time, and “single species” where each plant species were provided individually. Letters above box plots show results of pairwise comparisons for visit duration data. Red lines show the visitation rate (number of honey bee visits/hour) to each plant species for each experiment. Visit duration data were log10 transformed to achieve normality prior to analysis. We examined the effect of plant species on visit duration in an ANOVA using data from the single species experiment trials. We examined pairwise comparisons using Tukey contrasts (R library multcomp, functions glht and mcp). In a separate ANOVA using data from the single species and diversity trials, we examined the interaction effect of plant species and experiment (single species vs. diversity) on visit duration. (TIF) [file pone.0221800.s003.tif]
